# Precise Delineation and Transcriptional Characterization of Bovine Blood Dendritic-Cell and Monocyte Subsets

**DOI:** 10.3389/fimmu.2018.02505

**Published:** 2018-10-30

**Authors:** Stephanie C. Talker, Arnaud Baumann, G. Tuba Barut, Irene Keller, Rémy Bruggmann, Artur Summerfield

**Affiliations:** ^1^Institute of Virology and Immunology, Bern, Switzerland; ^2^Department of Infectious Diseases and Pathobiology, Vetsuisse Faculty, University of Bern, Bern, Switzerland; ^3^Interfaculty Bioinformatics Unit and Swiss Institute of Bioinformatics, University of Bern, Bern, Switzerland; ^4^Department for BioMedical Research, University of Bern, Bern, Switzerland

**Keywords:** dendritic cells, monocytes, transcriptome, cattle, cDC1, cDC2, pDC

## Abstract

A clear-cut delineation of bovine *bona fide* dendritic cells (DC) from monocytes has proved challenging, given the high phenotypic and functional plasticity of these innate immune cells and the marked phenotypic differences between species. Here, we demonstrate that, based on expression of Flt3, CD172a, CD13, and CD4, a precise identification of bovine blood conventional DC type 1 and 2 (cDC1, cDC2), plasmacytoid DC (pDC), and monocytes is possible with cDC1 being Flt3^+^CD172a^dim^CD13^+^CD4^−^, cDC2 being Flt3^+^CD172a^+^CD13^−^CD4^−^, pDC being Flt3^+^CD172a^dim^CD13^−^CD4^+^, and monocytes being Flt3^−^CD172a^high^CD13^−^CD4^−^. The phenotype of these subsets was characterized in further detail, and a subset-specific differential expression of CD2, CD5, CD11b, CD11c, CD14, CD16, CD26, CD62L, CD71, CD163, and CD205 was found. Subset identity was confirmed by transcriptomic analysis and subset-specific transcription of conserved key genes. We also sorted monocyte subsets based on their differential expression of CD14 and CD16. Classical monocytes (CD14^+^CD16^−^) clustered clearly apart from the two CD16^+^ monocyte subsets probably representing intermediate and non-classical monocytes described in human. The transcriptomic data also revealed differential gene transcription for molecules involved in antigen presentation, pathogen sensing, and migration, and therefore gives insights into functional differences between bovine DC and monocyte subsets. The identification of cell-type- and subset-specific gene transcription will assist in the quest for “marker molecules” that—when targeted by flow cytometry—will greatly facilitate research on bovine DC and monocytes. Overall, species comparisons will elucidate basic principles of DC and monocyte biology and will help to translate experimental findings from one species to another.

## Introduction

Dendritic (DC) and monocytic cells are bone-marrow derived innate immune cells with partly overlapping phenotypes and functions (1, 2). Both cell types are well-equipped with pattern-recognition receptors (3), with which they can rapidly sense infection, become activated, and consequently prime the adaptive immune system. Together with embryonically derived macrophages, DC and monocytes belong to the mononuclear phagocyte system and constitute clearly separate lineages as judged by ontogenetic studies in mice (4, 5) and single-cell transcriptomic analyses (6). As a consequence, *bona fide* DC need to be distinguished from monocyte-derived DC, which are DC-like cells that arise from activated monocytes in tissues.

The current view is that in all species *bona fide* DC can be classified into three subsets, each of them depending on different transcription factors for their development (7), and specialized to exert different functions (8). Classical/conventional DC type 1 (cDC1) and type 2 (cDC2) are specialized in initiation and shaping of T-cell responses. Mouse studies have provided evidence that cDC1 and cDC2 each preferentially activate CD8 T cells or different subsets of CD4 T cells, according to cross-presentation capabilities and cytokine repertoire (9, 10). The special feature of plasmacytoid DC (pDC), the third subset of DC, is the ability to rapidly produce large amounts of type I interferons upon activation (11), but pDC are also able to activate T cells and to cross-present antigen (12, 13).

Activated monocytes have also been reported to participate in T-cell priming and it is under debate whether they can be as efficient as *bona fide* DC in fulfilling this task (2). Monocytes can be divided into at least two subsets in mouse (14) and three subsets in humans (15), with different specializations in inflammation and tissue repair (16). Classical monocytes (cM) are defined as Ly6C/Gr1^+^ in mouse and CD14^+^CD16^−^ in humans, constitute the majority of blood monocytes, and were shown to function in tissue surveillance and antigen presentation, both under steady-state conditions and upon inflammation. In both human and mouse, cM were shown to express high levels of CCR2 and CD62L, and low levels of CX3CR1 (16). A smaller subset of non-classical monocytes (ncM), CD14^low^CD16^high^ in humans, and Ly6C/Gr1^−^ in mouse, was shown to patrol vessel walls and may preferentially give rise to “alternatively activated” monocyte-derived macrophages that function in tissue repair and regeneration (17, 18). These ncM were shown to express high levels of the chemokine receptor CX3CR1 and a number of adhesion molecules that enable them to crawl along endothelia (16). In humans, a third “intermediate” subset (CD14^high^CD16^+^) has been described containing a distinctive transcriptome (15). Also monocytes in cattle have been classified into three subsets according to their expression of CD14 and CD16 (19), similar to what has been described for humans (20). However, despite phenotypic similarities, several differences could be found regarding *in vitro* function of bovine and human monocyte subsets (21, 22).

The difficulty of studying *bona fide* DC lies in their low abundance (below 0.1% within PBMC of cattle) and in their phenotypic plasticity depending on tissue localization and inflammatory state (5). On top of this plasticity, DC have been shown to differ considerably between species, both in phenotype and function (23, 24), rendering direct translation of experimental findings difficult. Studying the genotype and phenotype of DC subsets in blood provides a snapshot of steady-state DC as they travel from the bone marrow to various lymphoid and non-lymphoid tissues, being as yet uninfluenced by cues from a particular tissue microenvironment.

While monocyte research in cattle is relatively young, some efforts have been made in the last two decades to functionally characterize bovine DC subsets, mainly in afferent lymph (25–31), but also in blood (30, 32–37). High expression of CD205 has been described to allow identification of DC in bovine afferent lymph (38). CD205^high^ afferent lymph DC (ALDC) of cattle have been classified into two subsets (27): a major subset being CD5^−^CD11a^−^CD13^−^CD26^−^CD172a^+^and a minor CD5^+^CD11a^+^CD13^+^CD26^+^CD172a^−^ subset. Within bovine PBMC, high expression of CD205 is also found on B cells (29), but two subsets of CD3^−^CD14^−^CD21^−^CD335^−^MHC^+^CD11c^+^ cDC in blood have been described, which can be discriminated based on the expression level of CD205 (39). Bovine pDC were postulated to be CD3^−^CD21^−^CD14^−^CD4^+^ and MHC-II^+^ (30), but were later described to be negative for MHC-II (35).

Thus, available data is in part conflicting and phenotypic definitions are still incomplete and not sufficiently elaborated to clearly differentiate all bovine DC subsets (23). Furthermore, recent developments in the field of DC research, enabled through the use of transcriptomics, have provided a great opportunity for a precise identification of subsets in veterinary species (24, 40). For example, transcription factors have been identified to be required for subset-specific development, and can therefore serve as key genes to confirm subset identity.

Consequently, the aim of the present study was to perform a precise delineation and comparative phenotypic and transcriptional characterization of bovine DC and monocyte subsets. To this end, we have performed extensive phenotypic characterization of *bona fide* Flt3^+^ DC and monocytes in blood of cattle, and have confirmed subset identity based on the transcription of subset-restricted key genes. Apart from subset identification, our transcriptomic analyses provide important insights into functional specialization, which is known to differ considerably between species.

## Materials and methods

### Isolation of bovine PBMC

Blood of cows (aged 2–6 years, Simmental, Holstein-Friesian, Red Holstein) was collected at the Clinic for Ruminants (Vetsuisse Faculty, University of Bern, Bern, Switzerland) or at the Institute of Virology and Immunology (IVI, Mittelhäusern, Switzerland) by puncturing the jugular vein. For sorting experiments and transcriptomic analyses, six different cows were used (*n* = 3 for sorting of DC subsets and *n* = 3 for sorting of monocyte subsets and cDC2′′). As an anticoagulant, citrate-based Alsever's solution (1.55 mM of C_6_H_12_O_6_, 408 mM of Na_3_C_6_H_5_O_7_·2H_2_O, 1.078 mM of NaCl, and 43 mM of C_6_H_8_O_7_, pH 6.2) was used. The blood sampling was performed in compliance with the Swiss animal protection law and approved by the animal welfare committee of the Canton of Bern, Switzerland, license number BE102/15. For PBMC isolation, blood was first centrifuged at 1,000 × g for 20 min. Then the buffy coat was collected and diluted with PBS to a ratio of 1 to 1 before being layered onto lymphocyte separation medium (1.077 g/ml; GE Healthcare Europe GmbH, Freiburg, Germany). After centrifugation (800 × g, 25 min), PBMC were collected and washed twice with cold PBS containing 1 mM EDTA (400 × g, 8 min). A final washing step was done at 250 × g (8 min) to remove platelets.

### Phenotyping of DC subsets by flow cytometry

Six-color phenotyping of DC subsets was performed in 96-well U-bottom microtiter plates with 2 × 10^7^ freshly isolated PBMC per sample. The staining encompassed five incubation steps, each for 20 min at 4°C. Washing steps between incubations were done with Cell Wash (BD Biosciences). Primary antibodies and secondary reagents are listed in Table 1. As a first step, PBMC were incubated with bovine IgG in order to block Fc receptors. ChromPure mouse IgG (Jackson ImmunoResearch) was used in the fourth step to block remaining binding sites of isotype-specific secondary antibodies. In the final step, anti-His-PE (Miltenyi Biotec) was added together with Live/Dead Near-IR stain (ThermoFisher) in order to stain biotinylated Flt3L. Bovine Flt3L (NCBI NM_181030.2) was produced as previously described (41) and employed to stain Flt3 expressing DC (42). Compensation was calculated by FACSDiva software following the measurement of single-stained samples. For each marker to be examined on DC subsets, a fluorescence-minus-one (FMO) control was included. Samples were acquired with a FACSCanto II flow cytometer (BD Biosciences) equipped with three lasers (405, 488, and 633 nm). At least 1.5 × 10^6^ cells were recorded in the “large-cell” gate.

**Table 1:** Antibodies and reagents used for flow cytometry.

	**Antigen**	**Clone/source of mAb**	**Detection/source**
**PANEL 1**			
Core	CD4	IL-A11/in house (ECACC)	Anti-IgG2a-PECy7/Southern Biotech
	CD13	CC81/Bio Rad	Directly conjugated to FITC
	CD172a	CC149/Bio Rad	Anti-IgG2b-Alexa647/Molecular Probes
	Flt3	n.a.	Anti-His-PE/Miltenyi
Phenotypic marker (Pm)	CD1	20.27/AdB Serotec	Anti-IgG1-biotin/Southern Biotech Streptavidin-BV421/BD Biosciences
	CD2	CC42/in house (ECACC)	
	CD5	CC29/in house (ECACC)
	CD14	CAM36A/Kingfisher
	CD26	CC69/Bio Rad
	CD40	IL-A156/Bio Rad
	CD62L	Du-1-29/in house (ECACC)
	CD80	IL-A159/Kingfisher
	CD86	IL-A190A/Kingfisher
	CD205	IL-A114/Bio Rad
	CD163	LND68A/Kingfisher
	BoLA-DRA	VPM54/in house (ECACC)
**PANEL 2**			
Core	CD4	CACT83B/Kingfisher	Anti-IgM-Alexa647/Southern Biotech
	CD13	CC81/AdB Serotec	Anti-IgG1-biotin/Southern Biotech Streptavidin-BV421/BD Biosciences
	CD172a	CC149/Bio Rad	Anti-IgG2b-Alexa488/Molecular Probes
	Flt3	n.a.	Anti-His-PE/Miltenyi
Pm	CD16	KD1/Bio Rad	Anti-IgG2a-PECy7/Southern Biotech
**PANEL 3**			
Core	CD4	IL-A11/in house (ECACC)	Anti-IgG2a-PECy7/Southern Biotech
	CD13	CC81/Bio Rad	Directly conjugated to FITC
	CD172a	DH59B/Kingfisher	Anti-IgG1-biotin/Southern Biotech Streptavidin-BV421/BD Biosciences
	Flt3	n.a.	Anti-His-PE/Miltenyi
Pm	CD11b	MM10A/Kingfisher	Anti-IgG2b-Alexa647/Molecular Probes
**PANEL 4**			
Core	CD4	IL-A11/in house (ECACC)	Anti-IgG2a-PECy7/Southern Biotech
	CD13	CC81/Bio Rad	FITC directly conjugated
	CD172a	DH59B/Kingfisher	Anti-IgG1-biotin/Southern Biotech Streptavidin-BV421/BD Biosciences
	Flt3	n.a.	Anti-His-PE/Miltenyi
Pm	CD11c	BAQ153A/Kingfisher	Anti-IgM-Alexa647/Southern Biotech
	CD71	IL-A77A/WSU
	CADM1	3E1/MBL	Directly conjugated to Alexa647

### Fluorescence-activated cell sorting (FACS) of DC subsets and monocyte subsets

For sorting of putative DC subsets, freshly isolated bovine PBMC were enriched for Flt3 expression by magnetic-activated cell sorting (MACS, Miltenyi Biotec) using His-tagged bovine recombinant Flt3L followed by anti-His-PE, and anti-PE magnetic beads (both Miltenyi Biotec). Enriched DC (~60-fold) were stained with anti-CD4, anti-CD13, and anti-CD172a and corresponding secondary antibodies anti-mouse IgG2a-PECy7, anti-mouse IgG1-Alexa488, and anti-mouse IgG2b-Alexa647 (see Table 1 for antibody reagents). ChromPure mouse IgG (Jackson ImmunoResearch) was used to block remaining binding sites of isotype-specific secondary antibodies. Then, putative pDC were sorted as Flt3^+^CD4^+^, putative cDC1 as Flt3^+^CD4^−^CD13^+^CD172a^low^, and putative cDC2′ as Flt3^+^CD4^−^CD13^−^CD172a^+^ using a FACS Aria (BD Biosciences).

In order to sort for monocyte subsets together with cDC2, a two-step staining was performed with 3 x 10^8^ freshly isolated PBMC. Cells were incubated with anti-CD14, anti-CD16, anti-CD172a and his-tagged bovine recombinant protein Flt3L, followed by anti-mouse IgM-Alexa647, anti-mouse IgG2a-PECy7, anti-mouse IgG2b-Alexa488, and anti-His-PE. CD172a^+^CD14^+^CD16^−^ (cM), CD172a^+^CD14^+^CD16^+^ (intM), CD172a^+^CD14^−^CD16^high^ (ncM) and CD172a^+^CD14^−^CD16^−^Flt3^+^ (cDC2′′) populations were sorted using a FACS Aria (BD Biosciences). All sorted subsets had a purity of at least 97%.

### RNA isolation and sequencing

FACS-sorted cell subsets were frozen to minus 80°C in TRIzol (ThermoFisher) for later RNA extraction. Total RNA was extracted using the Nucleospin RNA kit (Macherey Nagel), as recently described for porcine DC subsets (24). Quality and quantity of the purified RNA were assessed with an Agilent 2100 Bioanalyzer (Agilent Technologies) and a Qubit 2.0 Fluorometer (Life Technologies). Approximately 500 ng of high-quality RNA (RNA integrity number RIN>8) were used for non-directional paired-end mRNA library preparation (TruSeq Sample Preparation Kit; Illumina). Total mRNA libraries were randomly multiplexed in eight samples per lane and sequenced on the Illumina HiSeq3000 platform using 100 bp single-end sequencing. Between 25.2 and 41.1 million read pairs were obtained per sample. The reads were mapped to the bovine reference genome (Bovine Genome Database, UMD3.1) with Hisat2 v.2.1.0. FeatureCounts from Subread v.1.5.3 was used to count the number of reads overlapping with each gene, as specified in the Ensembl annotation (release 91). The RNAseq data are available in the European Nucleotide Archive (http://www.ebi.ac.uk/ena) under the accession number PRJEB28324.

### Interspecies subset comparison

Cell-type specific gene transcription signatures were compared with human, murine and porcine subsets. For DC subsets, microarray data from human (GEO GSE35457; blood CD141^+^ cDC1, blood CD1c^+^ cDC2, blood pDC, blood CD14^+^ monocytes) and mouse [GEO GSE35458; spleen CD8^+^ cDC1, spleen CD4^+^ cDC2, blood pDC, blood Gr1^high^ monocytes; Haniffa et al. (10)] and RNAseq data from pig [European Nucleotide Archive, accession number PRJEB15381; blood CD172^low^CADM1^+^ cDC1, blood CD172^high^CADM1^+^ cDC2, blood pDC, blood CD14^+^ monocytes; Auray et al. (24)] was used. For monocyte subsets, human [GEO GSE25913; CD14^++^CD16^−^, CD14^++^CD16^+^, CD14^+^CD16^+^; Wong et al. (43)], murine [GEO GSE17256; Gr1^high^, Gr1^low^; Ingersoll et al. (44)] and porcine [GEO GSE43898; CD163^high^, CD163^low^; Fairbairn et al. (45)] microarray data were used.

For each species, subtype-specific signatures were obtained by performing pairwise tests of differential gene transcription between each cell type and a pool of all remaining cell types. Tests were performed with DESeq2 v.1.18.1 for the RNA-seq data and GEO2R (NCBI) for the published microarray datasets. Microarray probes were excluded from further analysis if they measured multiple genes or if the ortholog of the gene in cattle could not be determined unambiguously. For genes measured by multiple probes on the array, only the one with the highest average transcription level was retained [following Miller et al. (46)]. All gene lists were sorted based on FDR-adjusted *P*-values to have the most highly upregulated genes at the top and the most strongly downregulated genes at the bottom.

Each cell-type specific signature from cattle was compared with all human, mouse and pig signatures using the R package OrderedList v.1.44.0 (47). This tool determines the number of shared elements in the tails of two lists and calculates a final similarity score where genes receive more weight the closer they are to the top or bottom of the list. This ensures the score is dominated by the genes showing the most significant differential transcription. We report similarity scores based on *n* = 1,000 genes each from the top and bottom of the lists. The relative similarity among the cell types was generally consistent for other values of *n* (assessed for values between 100 and 2,500). To assess the statistical significance of the similarity scores, the observed values were compared with a null distribution obtained by reshuffling the genes. Because invariant genes do not influence the similarity score, the middle 60% of genes were excluded from the permutations.

### Preparation of figures

Figures were prepared using FlowJo version 10 (FlowJo LLC, Ashland, OR), GraphPad Prism version 7.03 for Windows (GraphPad Software, San Diego, CA), R version 3.4.2, and Inkscape (www.inkscape.org).

## Results

### Phenotypic characterization of putative DC subsets in bovine blood

**Figure 1:**
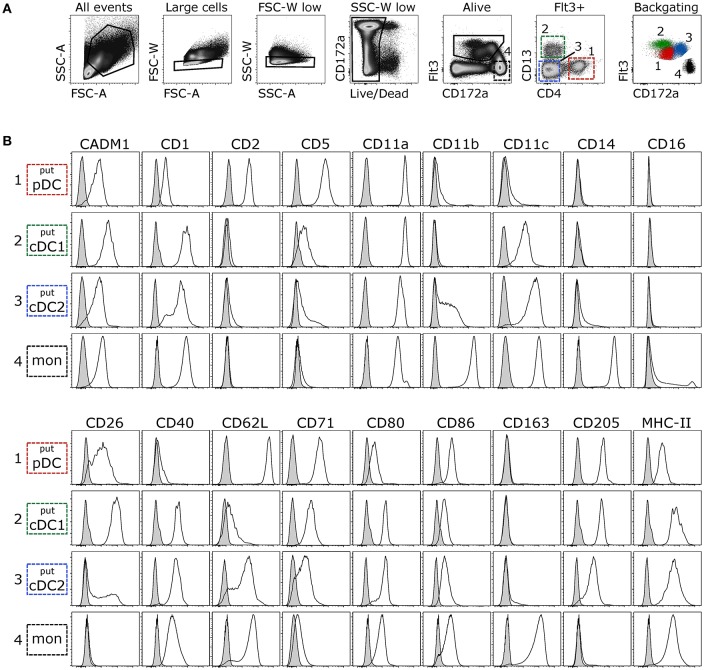
Phenotyping of putative DC subsets. Freshly isolated PBMC were stained for flow cytometry. **(A)** Large cells were selected in FSC-A vs. SSC-A and, following exclusion of doublets and dead cells, Flt3^+^ cells (DC) and Flt3^−^CD172a^high^ cells (monocytes) were gated. Within Flt3^+^ cells, three subsets were distinguished based on expression of CD4 and CD13. CD4^+^CD13^−^ putative pDC, CD4^−^CD13^+^ putative cDC1, and CD4^−^CD13^−^ putative cDC2. Backgating of the respective subsets illustrates expression levels of Flt3 and CD172a. **(B)** Empty histograms show the expression of various molecules on putative DC subsets and monocytes. Gray histograms show the FMO control. Data are representative for at least 3 animals.

Given that DC development and maintenance is dependent on signaling through the cytokine receptor Flt3 (48–50), and that this molecule was previously successfully used to identify porcine DC (24, 42), Flt3 was considered to be suitable for the identification of DC in bovine blood. Staining of bovine PBMC with the His-tagged Flt3L yielded a clearly defined population of binding cells, which expressed differing levels of CD172a (Figure 1A). Using antibodies against CD4 and CD13, this Flt3^+^ population could be further separated into three subsets. Based on previous studies with bovine DC (27, 30), the CD4^+^CD13^−^ subset was preliminarily defined as putative pDC, the CD4^−^CD13^+^ subset as putative cDC1, and the CD4^−^CD13^−^ subset as putative cDC2. Further phenotypic characterization of these subsets partially supported this classification and confirmed the identification of phenotypically distinct cell subsets (Figure 1B). In contrast to the vast majority of Flt3^−^CD172a^high^ monocytes, putative DC lacked monocyte-associated molecules CD14 and CD163 but expressed CD205 previously demonstrated to be expressed on bovine ALDC (25, 27, 51). CD11a was expressed on all DC subsets and on monocytes. CD16 was only expressed on a small proportion of monocytes and absent from DC. Molecules involved in antigen presentation and co-stimulation (CD1, CD40, CD80, MHC-II/BoLA-DRA) were expressed at higher levels on putative cDC than on putative pDC. The only exception was CD86, which was expressed to higher levels on putative pDC. CADM1 and CD26 showed the highest expression levels on putative cDC1, whereas monocyte-associated CD11b and CD11c were mainly expressed on putative cDC2. Finally, putative pDC stood out by their high expression of CD5, CD62L and CD71, and their exclusive expression of CD2.

### Confirmation of DC-subset identity and delineation from monocytes

**Figure 2:**
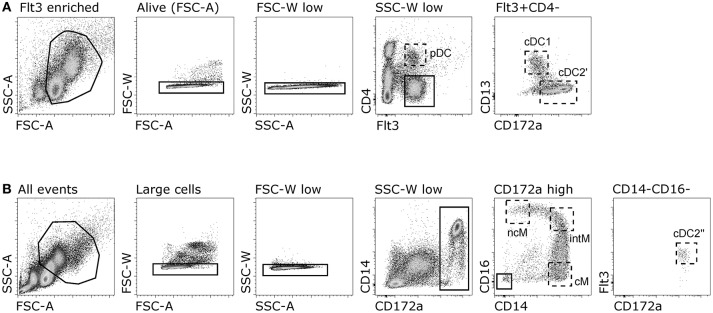
Gating strategy for sorting of DC and monocyte subsets. **(A)** Sorting of putative DC subsets. Flt3-enriched PBMC were gated for expression of Flt3 and CD4 (putative pDC), and Flt3^+^CD4^−^ cells were further gated as CD13^+^ (putative cDC1) and CD172a^+^CD13^−^ (putative cDC2′). **(B)** Sorting of monocyte subsets and putative cDC2. Whole PBMC were gated for large single cells expressing CD172a and monocyte subsets were gated as CD14^+^CD16^−^ (cM), CD14^+^CD16^+^ (intM), and CD14^−^CD16^+^ (ncM). Putative cDC2′′ were gated as Flt3^+^ within the CD14^−^CD16^−^ gate.

**Figure 3:**
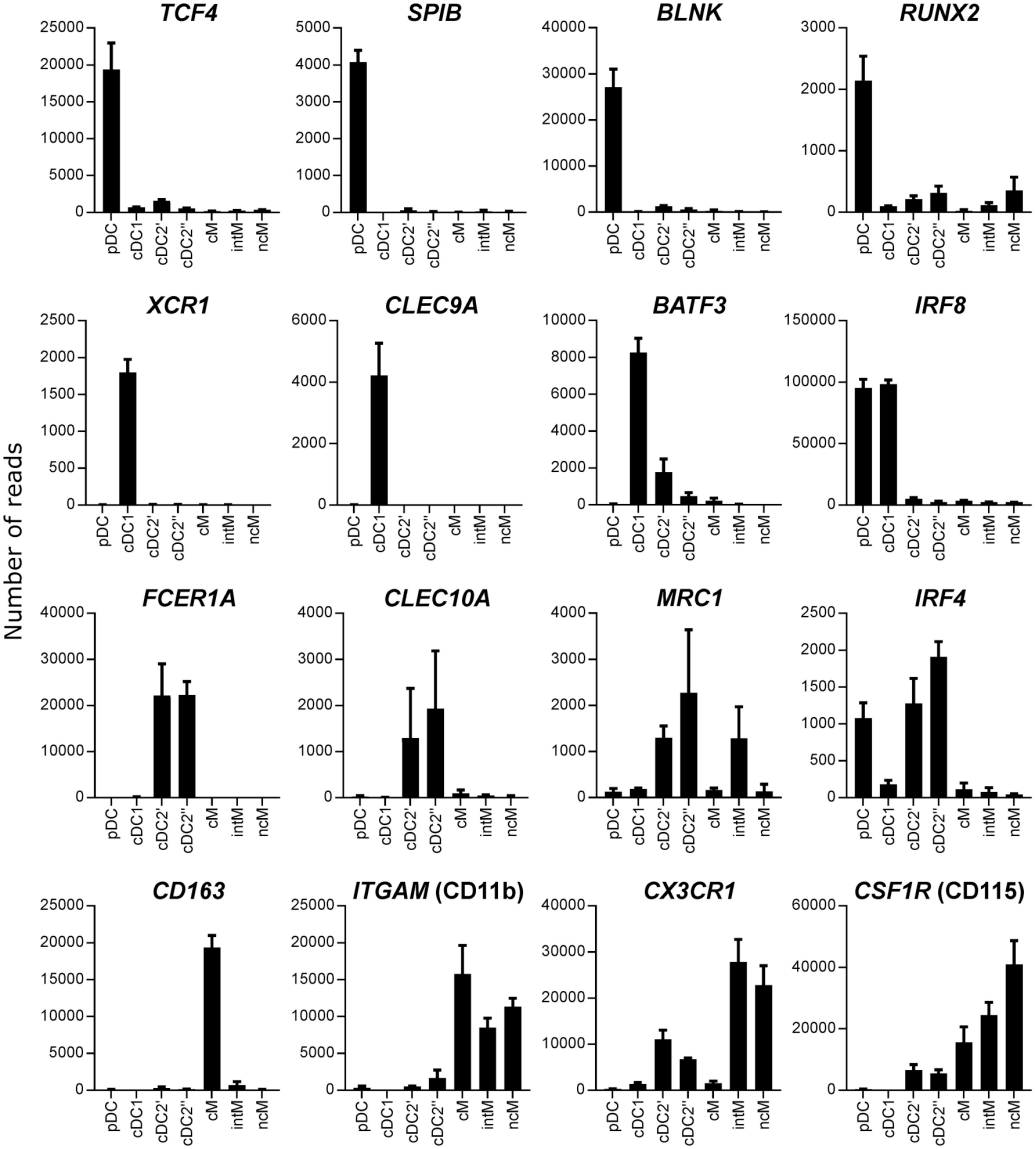
Transcription of key genes. High-throughput sequencing was performed on RNA isolated from sorted DC and monocyte subsets. Mean number of normalized reads and SD is shown for three animals and selected key genes.

As phenotypic analyses supported a correct identification of DC subsets, the next step was to confirm their identity by looking at subset-conserved gene transcription. For this purpose, Flt3^+^ CD4/CD13-defined subsets were sorted following magnetic enrichment for Flt3 expression (Figure 2A), and isolated RNA was subjected to high-throughput sequencing. Transcriptomic analysis revealed specific transcription of key genes that have been reported to be subset-defining in mouse, human, sheep and pigs (40). In fact, high levels of messenger RNA for *TCF4, SPIB, BLNK*, and *RUNX2* were exclusively found in putative pDC, *XCR1* and *CLEC9A* transcripts were only found in putative cDC1, and transcripts of *FCER1A* and *CLEC10A* were limited to putative cDC2 (Figure 3). *IRF4* transcripts were strongly enriched in pDC and cDC2, whereas *IRF8* was highly transcribed by pDC and cDC1. For the gene transcription data depicted in bar charts, a table with *p*-values for all pairwise comparisons performed with DESeq2 is provided as Supplementary File 1.

**Figure 4:**
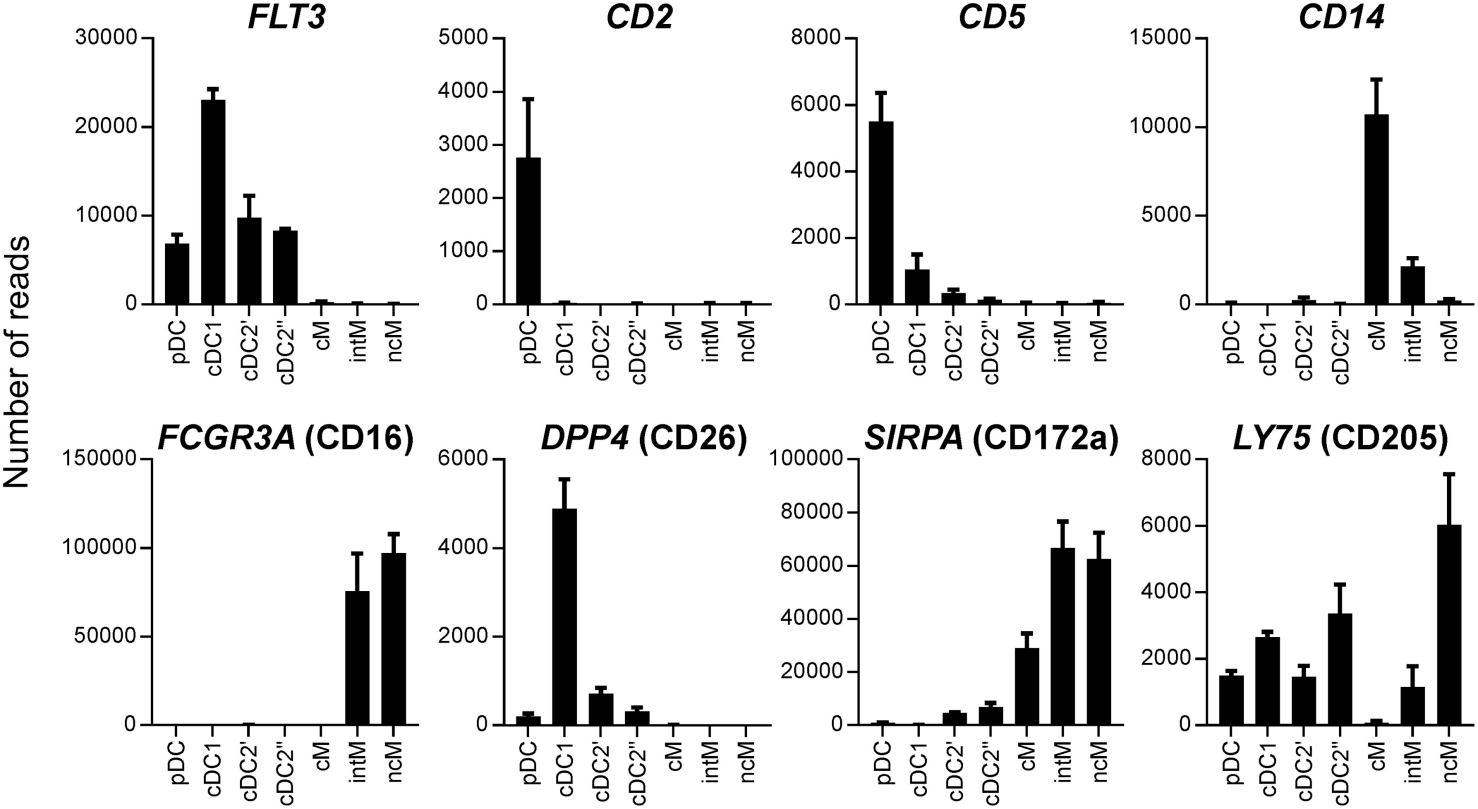
Transcript levels for molecules previously analyzed by flow cytometry. High-throughput sequencing was performed on RNA isolated from sorted DC and monocyte subsets. Mean number of normalized reads and SD is shown for three animals.

In all species, cDC2 are reported to phenotypically resemble monocytes, and the lack of *bona fide* cDC2-specific markers hampers their accurate identification, especially in tissues and under inflammatory conditions (52). In order to delineate the transcriptome of the newly identified DC subsets, especially cDC2, from monocytes, the three CD14/CD16-defined monocyte subsets reported for cattle (CD14^+^CD16^−^, CD14^+^CD16^+^, and CD14^−^CD16^+^) were sorted together with cDC2 (Figure 2B), and again their isolated RNA was subjected to high-throughput sequencing. Due to the proposed homology to human monocyte subsets (19), CD14^+^CD16^−^, CD14^+^CD16^+^, and CD14^−^CD16^+^ subsets were named classical (cM), intermediate (intM), and non-classical (ncM) monocytes throughout figures and text. The cDC2 subset sorted together with the monocyte subsets was labeled cDC2′′ to keep it apart from the cDC2 subset sorted together with pDC and cDC1, which was labeled cDC2′. The gating strategies for both sortings are shown in Figure 2. Transcriptomic data showed that monocytes lacked transcription of key genes associated with DC, and—as already shown by flow cytometry—monocytes contained transcripts for *ITGAM* (CD11b*)*. Transcription of *CD163* was limited to CD14^+^CD16^−^ cM. The chemokine receptor *CX3CR1* was found to be mainly transcribed by CD16^+^ monocytes, and to a lesser extent by cDC2 (Figure 3). Figure 4 illustrates transcript levels for additional molecules that have been analyzed by flow cytometry (Figure 1), and indicates overall good correlation of protein expression and mRNA content for monocytes and the different DC subsets.

**Figure 5:**
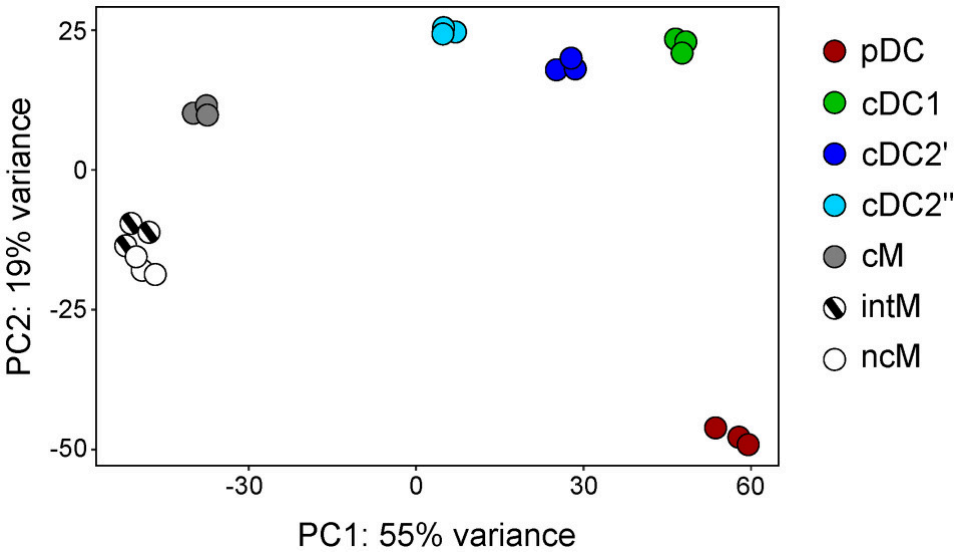
First two axes from a principal component analysis (PCA). High-throughput sequencing was performed on RNA isolated from sorted DC and monocyte subsets and the 500 most variable genes across all samples were included in the PCA. Each dot represents data of one individual animal.

Principal component analysis revealed a clear transcriptomic separation of cDC, pDC, and monocytes (Figure 5). Classical DC1 and cDC2 clustered most closely together but clearly formed separate clusters. Within monocytes, the two CD16^+^ subsets (intM, ncM) clustered together, but distinctively apart from CD14^+^CD16^−^ cM. To our surprise, the transcriptomes of cDC2′ and cDC2′′ show consistent differences (dark blue and light blue dots in Figure 5). When looking at the differentially transcribed genes between these two subsets, we found an enrichment in genes involved in translation and metabolism (Supplementary File 2). Despite this batch effect presumably caused by the different sorting approaches, Figures 3, 7–9 demonstrate that key genes and function-related genes, were transcribed with an almost identical pattern in cDC2′ and cDC2′′.

**Figure 6:**
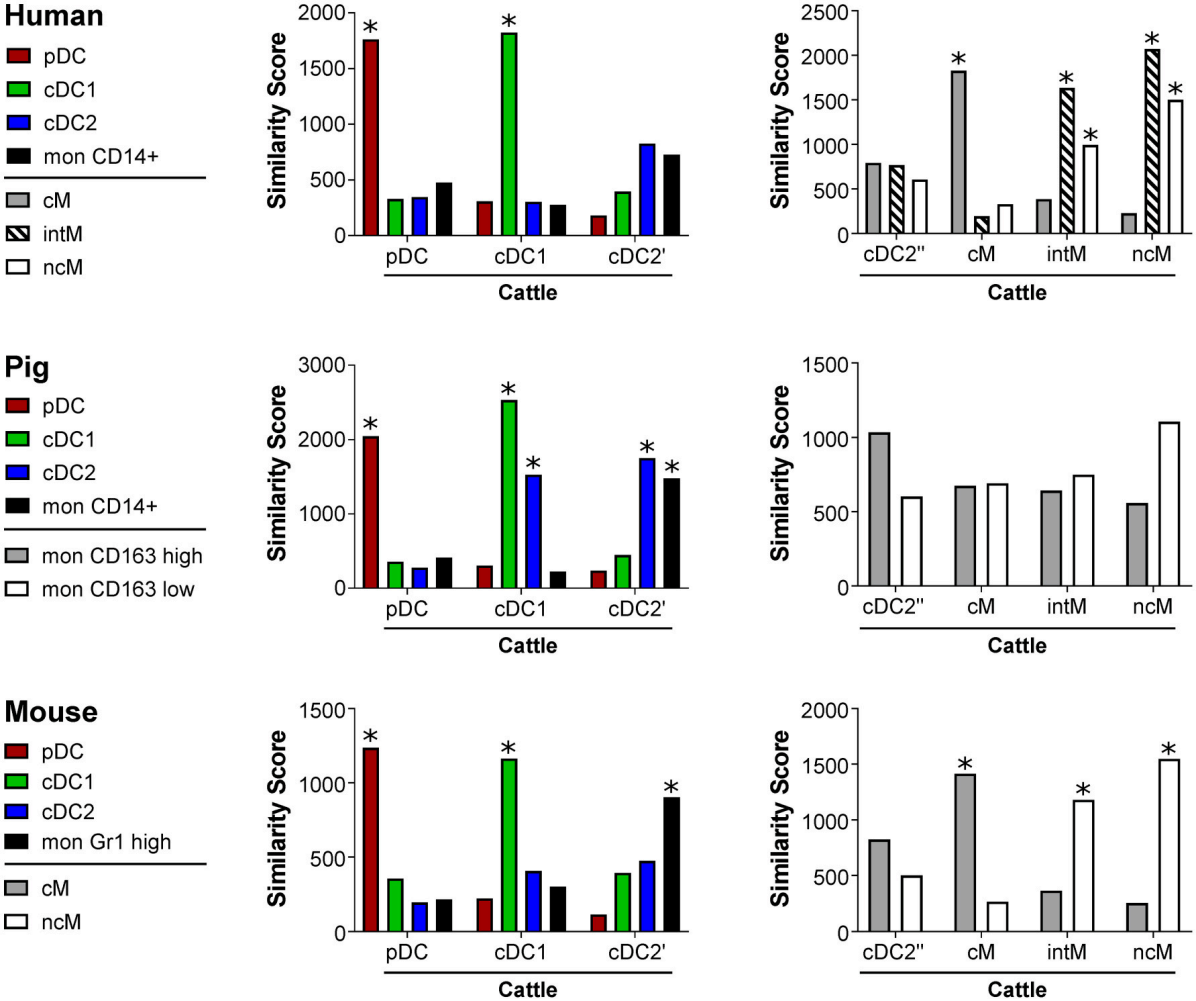
Similarity scores. Transcriptomic profiles of bovine DC and monocyte subsets were compared with signatures of corresponding human, porcine, and murine subsets. Statistical significance of the similarity scores was determined by comparing the observed values with a null distribution obtained by reshuffling the genes.*Empirical *p* < 0.001.

Cell-type specific gene transcription signatures of bovine subsets were compared to putative counterparts in human, pig and mouse by calculating similarity scores (Figure 6). For all species, high scores, indicating strong similarity, were obtained for pDC and cDC1. Bovine cDC2, however, also showed high similarity to porcine and murine monocytes. Bovine cM were found to be highly similar to human and murine cM. Bovine intM and ncM showed high similarity to human and murine intM and ncM. Nevertheless, both of these bovine subsets were most similar to human intM. For porcine monocyte subsets, previously defined based on differential CD163 expression (45), no significant similarity to either one of the bovine monocyte subsets could be found.

### Transcription of function-related genes in DC subsets and monocyte subsets

**Figure 7:**
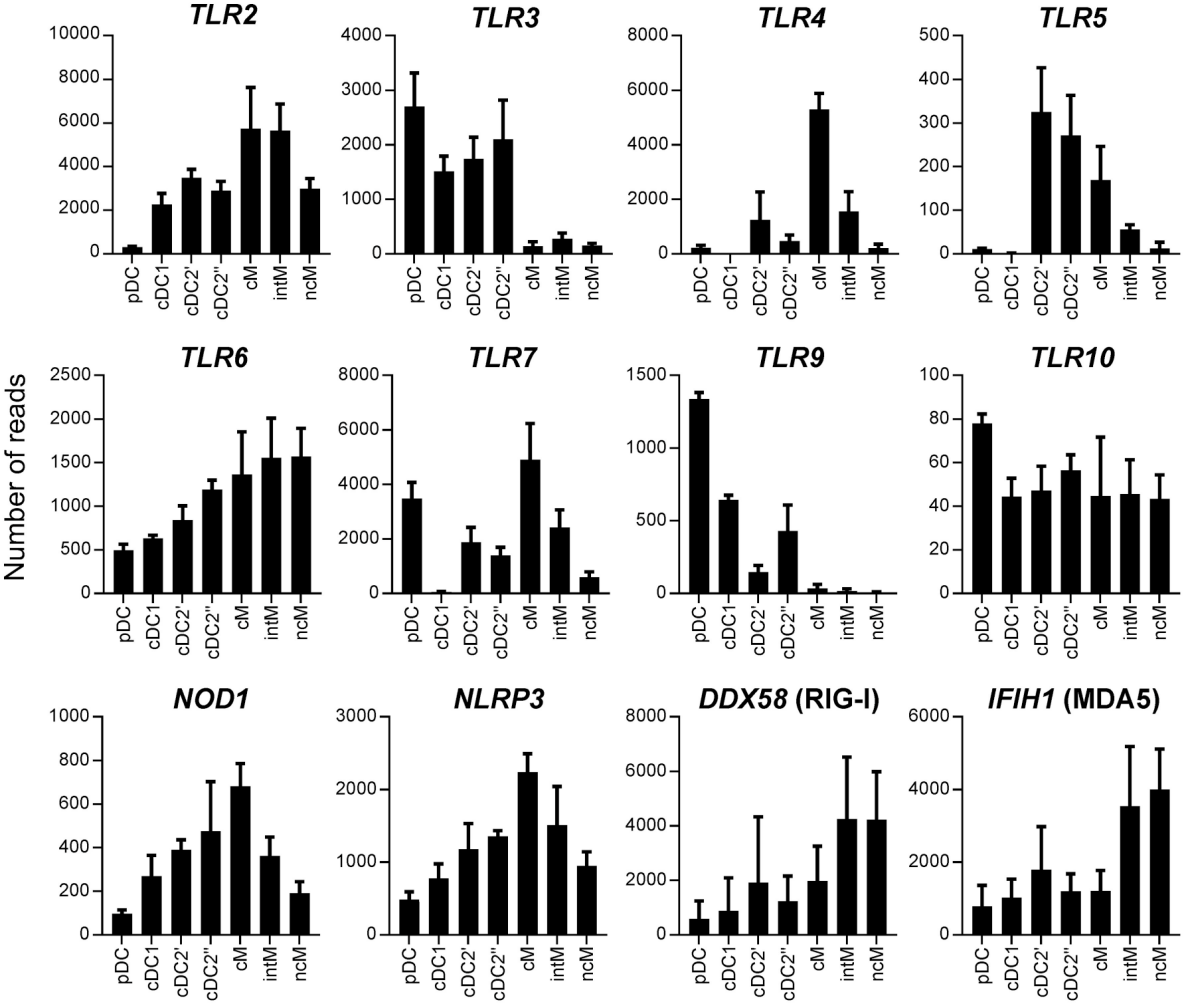
Transcription of pattern recognition receptor (PRR) genes. High-throughput sequencing was performed on RNA isolated from sorted DC and monocyte subsets. Mean number of normalized reads and SD is shown for three animals and selected PRR.

**Figure 8:**
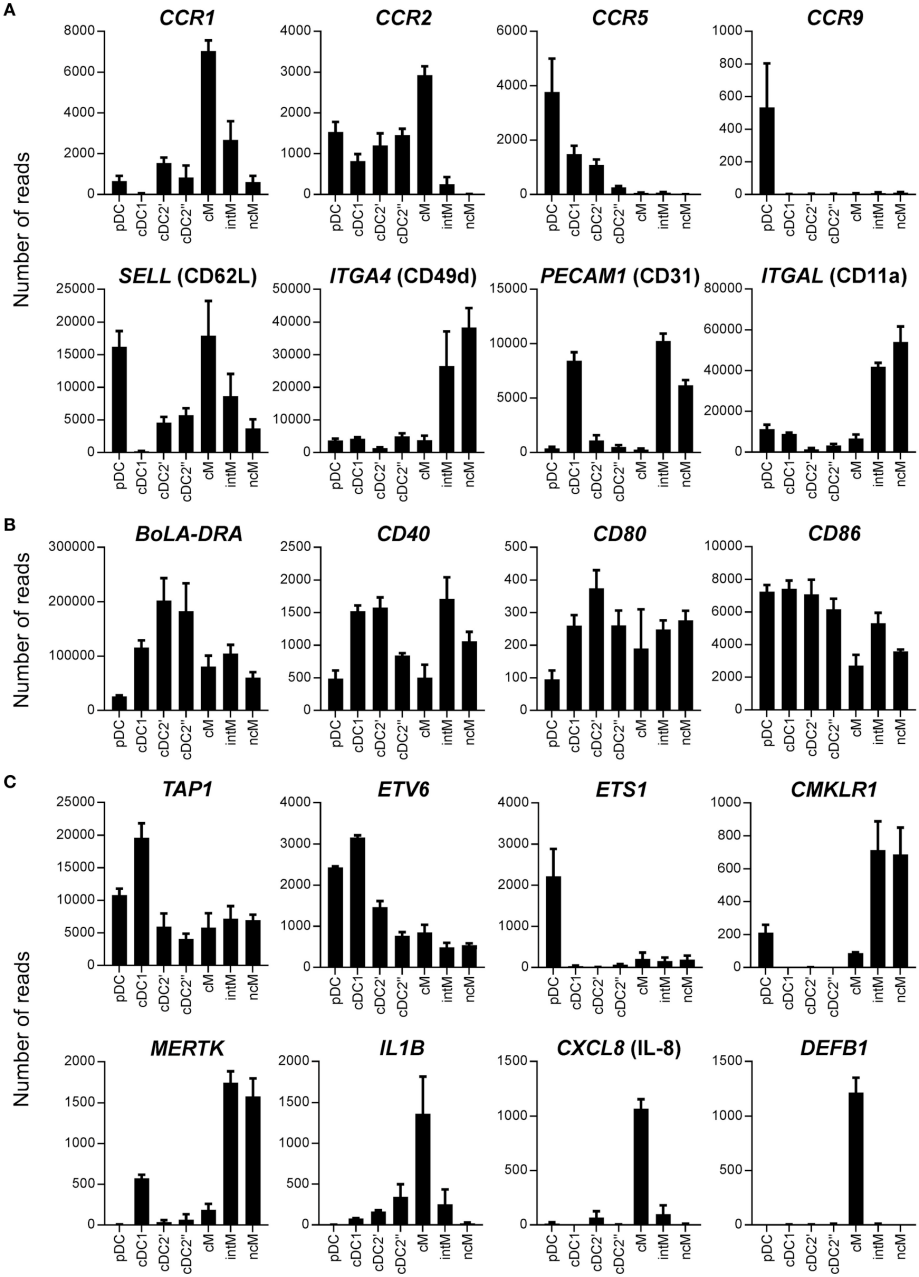
Transcription of genes coding for chemokine receptors and adhesion molecules **(A)**, BoLA-DRA and co-stimulatory molecules **(B)**, and miscellaneous function-related molecules **(C)**. High-throughput sequencing was performed on RNA isolated from sorted DC and monocyte subsets. Mean number of normalized reads and SD is shown for three animals and selected genes.

Transcriptomic data revealed pronounced differences between subsets in regard to gene transcription related to pathogen recognition, migration, antigen presentation and antimicrobial activity (Figures 7, 8). We found that pDC contained high levels of *TLR3, TLR7*, and *TLR9* transcripts, but showed very low transcription of *TLR2, TLR4*, and *TLR5*. Classical DC1 contained very low levels of *TLR4, TLR5*, and *TLR7* transcripts. All monocyte subsets almost completely lacked the transcription of *TLR3* and *TLR9*. Bovine cM showed high transcription rates of *TLR4* and *TLR7*, whereas ncM transcribed almost no *TLR4, TLR5*, and *TLR7*. *TLR10* transcription was found to be below 100 reads in all subsets. Cytosolic PRRs for detection of bacteria (*NOD1, NLRP3*) showed a high transcription rate in cM whereas *RIG-I* and *MDA5* for detection of viral RNA showed a high level of transcription in CD16^+^ monocytes (intM and ncM), though inter-individual variation was found to be high.

Differences between cell subsets were also observed in regard to transcription of genes mediating migration, extravasation, and recruitment to sites of infection (Figure 8A). Classical monocytes contained the highest levels of *CCR1* and *CCR2* transcripts. Non-classical monocytes lacked the transcription of *CCR2* and transcribed very low levels of *CCR1*, but together with intM they transcribed the highest levels of *CX3CR1* (Figure 3). *CCR5* gene transcription was highest in pDC and was found to be absent in all monocyte subsets. Interestingly, only pDC contained *CCR9* transcripts. *CD62L*, coding for an adhesion molecule mediating entry into secondary lymphoid organs through high endothelial venules (HEV), showed the highest transcription level in pDC and classical monocytes, and was only weakly transcribed in cDC1. Intermediate monocytes and ncM showed high transcription rates of *ITGA4, ITGB1, PECAM1, ITGAL*, and *ITGB2* (Figure 8A and data not shown). High *PECAM1* transcription was also found in cDC1. Regarding molecules involved in antigen presentation and co-stimulation (Figure 8B), pDC stood out among DC by showing by far the lowest levels of transcripts for BoLA-DRA, CD80, and CD40. Only *CD86* was transcribed in pDC to similar levels as in cDC. The highest levels of *BoLA-DRA* mRNA were found in the cDC2 subset. Overall, intM and ncM contained higher levels of transcripts for co-stimulatory molecules than cM. Within DC, *TAP1*, required for peptide-loading onto MHC-I molecules, showed the highest transcription in cDC1. Both dendritic cells and monocytes contained transcripts for ETV6, a transcription factor involved in IRF8-dependent development of cDC1, and only pDC showed high gene transcription for its antagonist ETS1. Only monocytes and pDC contained transcripts for the chemerin receptor CMKLR1, with the highest transcript levels detected in CD16^+^ monocytes. MERTK, involved in phagocytosis of dead cells, showed the highest gene transcription in intM and ncM, whereas classical monocytes contained the highest levels of transcripts for pro-inflammatory IL-1B, for neutrophil-attracting CXCL8 and the antimicrobial peptide DEFB1 (Figure 8C).

### Subset-specific gene transcription

Gene transcription specifically up- or downregulated in certain subsets can provide a deeper understanding of the specific biology and unique functions of a given subset. Data from pairwise comparisons was used to identify genes that are at least 5-fold up-or downregulated compared to all other subsets with a significance threshold of BH-adjusted *p* < 0.05. Intermediate and non-classical monocytes were treated as one subset, as were cDC2′ and cDC2′′. Genes that had been assigned below 200 reads in all subsets were omitted. Complete lists of subset-specific genes are provided as Supplementary File 3.

The highest number of differentially transcribed genes was found in pDC (230 upregulated, 83 downregulated). Many of the pDC-enriched genes are associated with B-cell development and function. Among the genes that were specifically downregulated in pDC were the inflammasome-associated genes *NLRC4* and *NLRP1* and semaphorin receptors *PLXND1* and *NRP2*. Also, *LGMN*, involved in processing of proteins for MHC-II presentation, showed significantly lower transcription in pDC. In cDC1, a total of 40 genes were upregulated (e.g., *CD84* and *CD103*) and 24 genes downregulated (e.g., *CD55*). For cDC2 (cDC2′ and cDC2′′), only transcripts for five genes were found to be exclusively upregulated and only *ECE1* was found to be at least 5-fold weaker transcribed compared to all other subsets. In cM, we found specific transcription of genes involved in phagocytosis (e.g., *DAB2, CLEC4D, CD163*) and antimicrobial activity (e.g., *DEFB1, DEFB3, HP, CHI3L1*). In total, 46 genes were up- and 6 genes downregulated in cM. Intermediate monocytes and ncM were treated as one subset, as—with the criteria mentioned above—no genes were found to be specifically transcribed when they were treated as separate subsets. Together, intM and ncM showed specific transcription of genes related to angiogenesis (e.g., *ACVRL1, PTPRB, GATA6*) and transcripts involved in the classical pathway of complement activation (*C1QA, C1QB, C1QC*), with 60 genes specifically up- and 7 genes specifically downregulated (Supplementary File 3).

**Figure 9:**
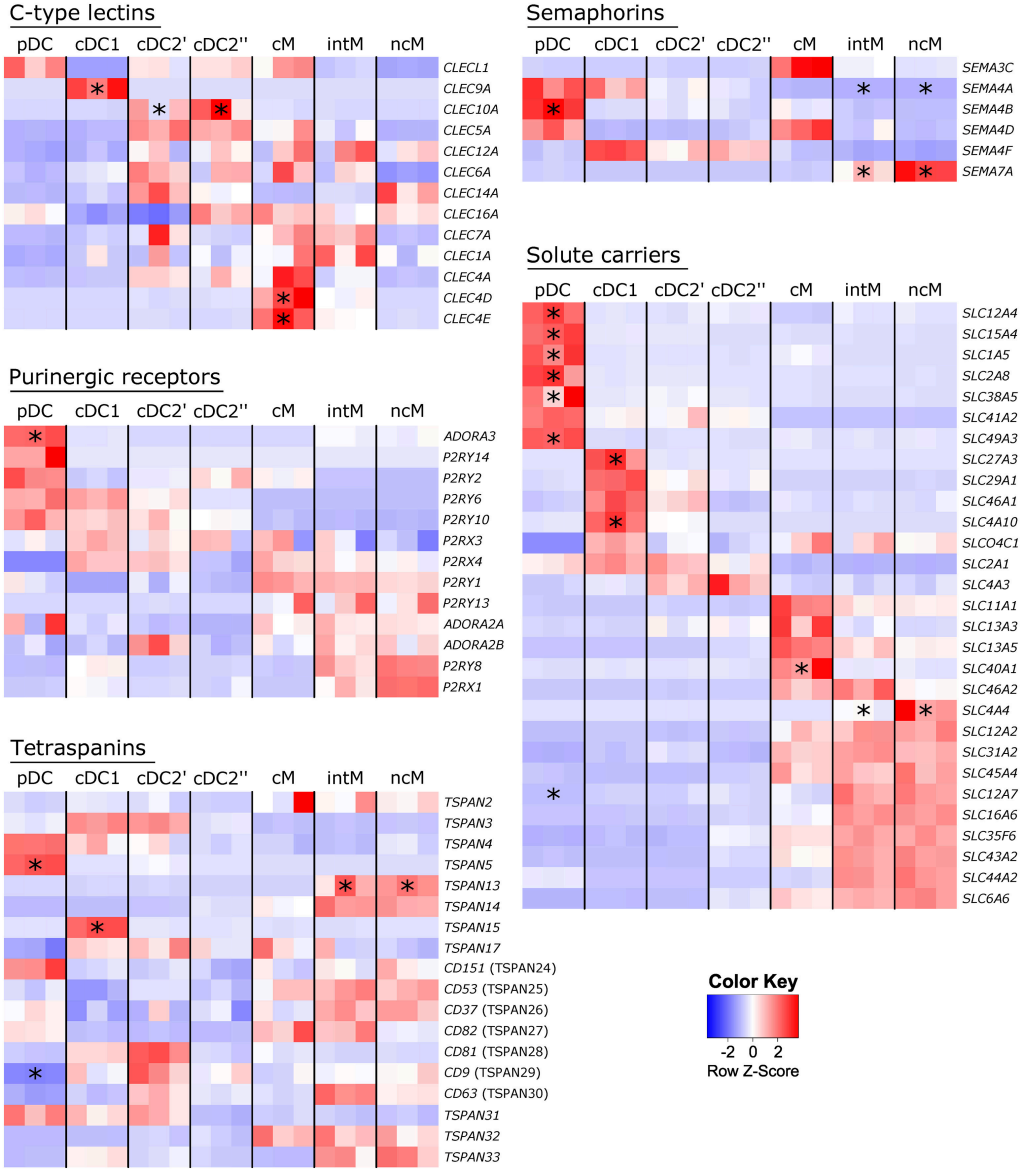
Subset-specific gene transcription. High-throughput sequencing was performed on RNA isolated from sorted DC and monocyte subsets. Read counts are displayed as heat maps for selected molecule classes. Asterisks indicate genes at least 5-fold up-/or downregulated in certain subsets (*p* < 0.05) as compared to all other subsets. Intermediate and non-classical monocytes were treated as one subset, as were cDC2′ and cDC2′′. Complete lists of at least 5-fold up-/or downregulated genes are provided as Supplementary File 3.

Molecule classes repetitively found to be differentially transcribed across subsets and reported to be relevant for immune responses are illustrated as heat maps in Figure 9 and encompass C-type lectins, purinergic receptors, tetraspanins, semaphorins and solute carriers. Plasmacytoid DC specifically contained transcripts of *ADORA3, TSPAN5, SEMA4B* and six different solute carrier genes. Transcription of genes coding for C-type lectins was found to be specific not only for cDC1 (*CLEC9A*) and cDC2 (*CLEC10A*), but also for cM (*CLEC4D, CLEC4E*). Transcripts of *SEMA7A* were specifically enriched in intM and ncM, whereas *SEMA4A* transcription was found to be specifically downregulated in these two monocyte subsets.

The subset-specific transcription patterns of these genes allow insights into functional specialization of subsets, which, when conserved across species, may be of particular relevance for subset-specific contributions to immune responses.

## Discussion

The present study provides a phenotypic definition of pDC, cDC1, and cDC2 in blood of cattle, which could be confirmed by subset-restricted transcription of key genes. These key genes, often required for the development and differentiation of certain subsets, have proved to be highly conserved across species (23, 40, 53) and have therefore served to validate subset identity in other species such as human (52, 54, 55), pig (24), sheep (56, 57), and horse (41). Our data strongly suggest that pDC and cDC1 in blood of cattle can be identified by the relatively simple phenotype Flt3^+^CD4^+^CD13^−^ and Flt3^+^CD4^−^CD13^+^, respectively. The identification of cDC2 remains challenging in all species due to their phenotypic similarity to monocytes, and the paucity of cDC2-specific key genes. We therefore directly compared the transcriptome of monocyte subsets and putative cDC2. Accordingly, we employed two different gating strategies for cDC2. One based on the expression of Flt3 and the exclusion of pDC and cDC1, that is Flt3^+^CD4^−^CD13^−^, and the other based on the expression of Flt3 and CD172a, and the exclusion of monocytes, that is Flt3^+^CD172a^+^CD14^−^CD16^−^. Both putative cDC2 populations contained high levels of *FCER1A* transcripts and transcribed *IRF4* in the almost complete absence of *IRF8* transcripts, supporting their correct identification (52). Moreover, both cDC2 subsets specifically transcribed the gene for CLEC10A, a molecule recently shown to be specific for human cDC2 (58). The separation of the two cDC2 subsets in the PCA was found to be mainly due to differences in housekeeping genes, and not in genes related to lineage or function. It is possible, however, that—in addition to the batch effect introduced by differences in sample handling—the different gating strategies have led to the sampling of sub-subsets within cDC2, especially in regard to expression levels of CD172a (see Figure 2 for a comparison of the two gating strategies). This only highlights the difficulty of phenotypically defining cDC2, which—in addition to their similarity to monocytes—are known to be heterogeneous (52).

Bovine DC subsets have been addressed in numerous studies, looking at blood (30, 32–37, 39), afferent lymph (25–31), lymph nodes (30), spleen (59), skin (29) and intestine (60). Sei et al. (35) described three DC subsets in blood of cattle after depletion for CD3, IgM, CD14, and CD11b, defining pDC as CD4^+^MHC-II^−^, and two subsets of cDC as being CD4^−^MHC-II^+^ and either positive or negative for CD11c. However, our data clearly show that pDC can express MHC-II and that both subsets of cDC express CD11c, with slightly higher expression on cDC2. In another study, a distinction between two cDC subsets in bovine blood was based on high and low expression of CD205 on cells negative for CD3, CD14, CD21, and CD335, and positive for MHC-II and CD11c (39). Indeed, we found lower levels of CD205 expression on cDC2, which may correspond to the CD205^low^ subset described in this study, but our data indicate that the discrimination of blood DC based on CD205 is difficult. Furthermore, in contrast to this study, we could not find differences in forward scatter and CD86 expression between cDC1 and cDC2. Recently, Li et al. (36) followed a complicated protocol of depleting for CD3, CD11b, CD14, CD21, and CD335, and enriching for CD26 to get hold of putative cDC1 in bovine blood, which were enriched in *XCR1* and *CLEC9A* mRNA. Their findings on the phenotype of cDC1 are in line with our study, but according to our data, the proposed phenotype of CD26^+^CADM1^+^CD205^+^MHC-II^+^CD11b^−^ is not suited to unequivocally delineate cDC1 from cDC2 and pDC. Our data demonstrate that in the peripheral blood of cattle neither CD26, CD205 nor CADM1 are expressed in a subset-restricted manner. Afferent lymph DC of cattle have been classified into a major subset being CD5^−^CD11a^−^CD13^−^CD26^−^CD172a^+^and a minor CD5^+^CD11a^+^CD13^+^CD26^+^CD172a^−^ subset (27). These subsets phenotypically resemble cDC2 and cDC1 we identified in bovine blood. In contrast to ALDC (25), all blood DC expressed CD11a, though transcriptomic data revealed even higher transcript levels in intM and ncM (2,000 reads in cDC2 vs. 40,000 reads in CD16^+^ monocytes).

Gibson et al. (34) could detect IFN type I responses to CpG stimulation in bovine PBMC depleted for CD14, CD3, CD2, CD4, CD8, CD21, and IgM, and suspected to have enriched bovine pDC. Also, this study appears to be in conflict with our data which demonstrates CD4 and CD2 expression on bovine pDC. Functional studies on bovine mononuclear phagocyte responses to CpG are required to understand the reasons for this discrepancy.

Due to a stringent gating strategy based on Flt3 expression, a contamination with monocytes in the cDC2 gate is unlikely in both cases, however it cannot be ruled out that DC precursors are included in our current definition of cDC2. In support of this, we found transcription of *AXL* in bovine cDC2, a gene associated with a DC precursor subset recently detected in human blood by single-cell sequencing of lineage^−^HLA-DR^+^ cells (6). Clearly, single-cell transcriptomics performed in the future will help to decipher the full spectrum of DC heterogeneity in cattle.

The transcriptomic data at hand can give valuable insights into functional potential of bovine DC and monocyte subsets under steady-state conditions. Data obtained so far from human, mouse, pig and cattle point toward species-specific differences in DC and monocyte functions (19, 23, 24, 44). The propagated importance of pDC for early sensing of viral infection is supported by the high transcription rate of *TLR3, TLR7*, and *TLR9* found in bovine pDC. Notably, *TLR3* transcription is neither found in murine nor in human pDC (61, 62), but has only recently been described for porcine pDC (24). The apparent lack of *TLR4* and *TLR7* transcription in cDC1 is in line with observations from bovine ALDC, where cDC1 were shown to contain significantly less *TLR4* and *TLR7* mRNA as compared to certain subsets of cDC2 (31).

The high expression of *CD62L* in bovine pDC and cM, also on protein level, suggests that bovine pDC and cM can enter secondary lymphoid tissue via HEV in steady state, as is reported for murine pDC and monocytes (63–65). Expression of *CMKLR1* and *CCR5* in bovine pDC is likely to assist lymph-node entry through HEV (63, 66). Steady-state entry into lymph nodes may be important in regard to peripheral tolerance induction (67, 68). In terms of central tolerance induction, *CCR9* expression by pDC has gained attention (69), as it was shown to mediate migration of antigen-bearing pDC to the thymus. We found *CCR9* to be exclusively transcribed in pDC, however due to the lack of a specific mAb, we could not confirm CCR9 expression on protein level. Murine pDC have been shown to optimize cDC1 maturation and cross-presentation in lymph nodes (70). In this regard, CCR5 was shown to be vital for migration of pDC toward clusters of cDC1 and activated CD8 T cells. The high transcription of *CCR5* and *CD62L* in bovine pDC may point toward a similar function in lymph nodes of cattle. The high expression of CD71 (transferrin receptor) we observed on bovine pDC in comparison to the other DC subsets has also been described for murine pDC in tissues and might be related to high iron demand of pDC upon rapid and large-scale production of type I interferons (71).

Like their murine and human counterparts (57), bovine cDC1 specifically transcribe *CLEC9A*, an endocytic receptor mediating cross-presentation of antigens derived from apoptotic cells (72). This, together with high transcription of *TAP1*, points toward a prominent role of cDC1 in cross-presentation and CD8 T-cell activation, as has been shown for murine cDC1 (73). In support of this, bovine cDC1 transcribe *ETV6* in the absence of *ETS1* transcription, which was shown to be essential for optimal development of cross-priming function in murine cDC1 (74).

Bovine cDC1 share a high transcription of *PECAM1* with intM and ncM. For murine DC, *PECAM1* (CD31) has been shown to function as a co-inhibitory receptor favoring tolerogenic responses (75). Similarly, we found *MERTK*, which—in human DC—has been described to act as a negative regulator of T-cell activation (76), to be transcribed mainly in cDC1 and CD16^+^ monocytes. The biological relevance of regulatory *PECAM1* and *MERTK* expression in cDC1 may be linked to their ability to evoke potentially harmful CD8 T cell responses that need to be kept under control.

In mouse and human, cDC2 have been reported to preferentially activate CD4 T cells (9, 10). While bovine cDC1 and cDC2 expressed similar levels of surface BoLA-DRA protein, the content of BoLA-DRA mRNA was found to be significantly higher in cDC2. This may suggest higher levels of intracellularly stored BoLA-DRA protein in cDC2, which would argue for their specialization in CD4 T-cell activation also in cattle.

In various species, such as mouse and human (16) and pigs (45), monocytes in peripheral blood have been split into different subsets according to phenotype and function. The CD14^+^CD16^−^ monocyte subset in cattle contained the highest levels of transcripts for chemokine receptors mediating entry into inflamed tissues (CCR1, CCR2) and also expresses high levels of CD62L, allowing this subset to access lymph nodes directly from the blood via HEV, as reported for murine cM under inflammatory and steady-state conditions (65, 77). Also, they are phenotypically very close to human CD14^+^CD16^−^ cM (19). In humans, another distinction has been made between CD16^+^ monocytes expressing CD14 (intermediate) and monocytes almost lacking CD14 expression (non-classical). Analogous to the human subsets, we have also sorted intM and ncM, however they were found to be very similar in their transcriptomic profile, as determined by PCA, and may therefore not represent functionally distinct subsets in cattle. Our data demonstrate that CD16^+^ monocytes (intM and ncM) in cattle most probably resemble Ly6C/Gr1^−^ monocytes in mice, which were suggested to preferentially give rise to alternatively activated macrophages that contribute to tissue regeneration (17, 18, 78). Similar to ncM of human, mouse, rat, and pig (79), bovine CD16^+^ monocytes contain high levels of *CX3CR1* transcripts and little or no transcripts for *CCR2* and *CD62L*. Also, bovine CD16^+^ monocytes selectively transcribe high levels of adhesion molecules *ITGA4, PECAM1* and *ITGAL*, indicating that—also in cattle—CD16^+^ monocytes adhere to the endothelium as a marginal pool. In support of their function in tissue regeneration, bovine CD16^+^ monocytes specifically transcribe genes involved in angiogenesis, as has been reported for human CD14^+^CD16^+^ monocytes (15). Also, the genes coding for PECAM1, associated with high angiogenic capacity of human monocytes (80), MERTK, described to mediate phagocytosis of apoptotic cells (efferocytosis) by human intM (81), and GAS6, reported to be specifically secreted by anti-inflammatory human macrophages (81), are selectively transcribed by bovine CD16^+^ monocytes. As is the gene for C1q, described to mediate efferocytosis and to have pro-angiogenic functions (82).

Porcine CD163^+^CD14^−^ monocytes were suggested to be the equivalent of ncM of human and mouse, as they transcribe low levels of *CCR2* and high levels of *CX3CR1* (83). However, the transcriptomic profile of bovine ncM was found to be more similar to the porcine CD163^low^ monocytes. When compared to the bovine data, it seems like CD163 is not able to discriminate between porcine cM and ncM, even if some similarities in gene transcription have been found between CD163^+^ porcine monocytes and CD16^+^ human monocytes (45). Taken together, also in cattle, the task of microbial combat seems rather to be taken by cM, which transcribe a whole array of antimicrobial genes and seem to be ideally equipped for an inflammatory response to bacterial infection, reflected by their gene transcription of TLRs, NOD-1 like receptors, and defensins.

Other genes found to be selectively transcribed by DC and monocyte subsets code for C-type lectins, purinergic receptors, tetraspanins, semaphorins, and solute carrier proteins. The most striking selectivity of transcription was found for proteins of the solute carrier (SLC) family, which seems to be—at least in part—conserved across species. *SLC11A1* is also expressed in human and murine myeloid cells as well as in bovine, human, and murine innate lymphocytes, where *SLC11A1* expression was shown to promote cell activation (84, 85, 86). SLC15A4, selectively transcribed in bovine and also in human (6) pDC, was shown to be required for signaling through TLR7 and TLR9 in murine pDC (87) and, as a consequence, for pDC-mediated control of persistent viral infection (88). Although a subset-specific expression of transporter proteins has been observed some time ago (89), the role of most SLC proteins in DC or monocyte biology remains elusive and needs further investigation.

Purinergic receptors can evoke pro- and anti-inflammatory immune responses when binding extracellular nucleotides released by cellular stress or apoptosis (90). Signaling via P2Y receptors was shown to negatively regulate IFN-α production in human pDC (91) and it will be interesting to see whether the prominent transcription of P2Y receptors in bovine pDC serves a similar function.

In mice, the tetraspanins CD82 and CD37 have been described to be involved in migration and antigen presentation of DC (92). Non-activated bone marrow-derived DC (BMDC) were shown to be CD37^high^CD82^low^, whereas late activated BMDC showed the inverse phenotype, with a migratory, cytoskeletal and antigen presentation machinery optimized for activating naïve T cells. Steady-state bovine classical DC completely lack transcription of *CD82*, but clearly contain mRNA of *CD37* (on average 2,000 reads), suggesting that the findings in mice might be transferable to cattle. For the tetraspanins CD9, CD37, CD63, CD81, CD82, and CD151 it is suggested that they play an important role in regulating the function of DC surface receptors (93). In agreement with murine pDC, bovine steady-state pDC lack the expression of *CD9*, a tetraspanin that is otherwise broadly expressed in leukocytes and was shown to function in the stabilization of the immunological synapse (94).

Semaphorins play important roles in guiding immune responses (95). We found certain semaphorins to be selectively transcribed by DC and monocyte subsets. SEMA4B has been described to suppress basophil-mediated Th2 skewing (96). As bovine pDC selectively transcribe high levels of *SEMA4B*, it may be speculated that they assist SEMA4B-producing T cells in regulating basophil responses. *SEMA4A* transcripts were enriched in both bovine pDC and cDC1. SEMA4A has been shown to induce activation and differentiation of T cells into antigen-specific subsets (97), and to support Treg function and survival (98). Also SEMA4D, found to be exclusively transcribed in bovine pDC and cM, was found to enhance B-cell activation (99) and to promote DC maturation and T-cell responses (100). Finally, SEMA7A, selectively transcribed by bovine CD16^+^ monocytes, has been described as a potent autocrine activator of human monocytes (101), however the pro-inflammatory phenotype evoked by SEMA7A is somewhat in contradiction to the rather anti-inflammatory role proposed for bovine CD16^+^ monocytes.

In summary, a clear-cut identification of bovine DC and their delineation from monocytes, as presented here, provides the basis for future studies on their functions and their involvement in disease pathogenesis, which will enable a compilation of strategies to improve vaccines and immunotherapeutics. This work also represents the basis for future work addressing transcriptomic signatures and functions during infection and inflammation, which includes different lymphoid and non-lymphoid tissues. In addition, comparative studies on DC function in mammals provide a deeper understanding of DC biology in general, as they reveal conserved and species-specific characteristics of DC subsets.

## Ethics statement

The animal experiments were performed according to the local law and were approved by the Ethical Committee for Animal Experiments of the Canton of Bern.

## Author contributions

ST performed laboratory work, analyzed the data, and wrote the manuscript. AB and GB performed laboratory work and analyzed data. IK and RB performed bioinformatic analyses. AS designed and supervised the overall project.

### Conflict of interest statement

The authors declare that the research was conducted in the absence of any commercial or financial relationships that could be construed as a potential conflict of interest.

## Supplementary material

Supplementary File 1 P values for pairwise comparisons performed with DESeq2.

Supplementary File 2 GO Term Enrichment Analysis cDC2′ vs. cDC2″.

Supplementary File 3 Subset-specific genes.

## References

[1] Merad M Sathe P Helft J Miller J Mortha A. The dendritic cell lineage: ontogeny and function of dendritic cells and their subsets in the steady state and the inflamed setting. Annu Rev Immunol. (2013) 31:563–604. 10.1146/annurev-immunol-020711-074950 23516985 PMC3853342

[2] Jakubzick CV Randolph GJ Henson PM. Monocyte differentiation and antigen-presenting functions. Nat Rev Immunol. (2017) 17:349–62. 10.1038/nri.2017.28 28436425

[3] Akira S Uematsu S Takeuchi O. Pathogen recognition and innate immunity. Cell (2006) 124:783–801. 10.1016/j.cell.2006.02.015 16497588

[4] Geissmann F Manz MG Jung S Sieweke MH Merad M Ley K. Development of monocytes, macrophages, and dendritic cells. Science (2010) 327:656–61. 10.1126/science.1178331 20133564 PMC2887389

[5] Guilliams M Ginhoux F Jakubzick C Naik SH Onai N Schraml BU. Dendritic cells, monocytes and macrophages: a unified nomenclature based on ontogeny. Nat Rev Immunol. (2014) 14:571–8. 10.1038/nri3712 25033907 PMC4638219

[6] Villani AC Satija R Reynolds G Sarkizova S Shekhar K Fletcher J. Single-cell RNA-seq reveals new types of human blood dendritic cells, monocytes, and progenitors. Science (2017) 356:eaah4573. 10.1126/science.aah4573 28428369 PMC5775029

[7] Murphy TL Grajales-Reyes GE Wu X Tussiwand R Briseno CG Iwata A. Transcriptional control of dendritic cell development. Annu Rev Immunol. (2016) 34:93–119. 10.1146/annurev-immunol-032713-120204 26735697 PMC5135011

[8] Mildner A Jung S. Development and function of dendritic cell subsets. Immunity (2014) 40:642–56. 10.1016/j.immuni.2014.04.016 24837101

[9] Schlitzer A Ginhoux F. Organization of the mouse and human DC network. Curr Opin Immunol. (2014) 26:90–9. 10.1016/j.coi.2013.11.002 24556405

[10] Haniffa M Collin M Ginhoux F. Ontogeny and functional specialization of dendritic cells in human and mouse. Adv Immunol. (2013) 120:1–49. 10.1016/b978-0-12-417028-5.00001-6 24070379

[11] Swiecki M Colonna M. The multifaceted biology of plasmacytoid dendritic cells. Nat Rev Immunol. (2015) 15:471–85. 10.1038/nri3865 26160613 PMC4808588

[12] Hoeffel G Ripoche AC Matheoud D Nascimbeni M Escriou N Lebon P. Antigen crosspresentation by human plasmacytoid dendritic cells. Immunity (2007) 27:481–92. 10.1016/j.immuni.2007.07.021 17869134

[13] Villadangos JA Young L. Antigen-presentation properties of plasmacytoid dendritic cells. Immunity (2008) 29:352–61. 10.1016/j.immuni.2008.09.002 18799143

[14] Geissmann F Jung S Littman DR. Blood monocytes consist of two principal subsets with distinct migratory properties. Immunity (2003) 19:71–82. 10.1016/S1074-7613(03)00174-2 12871640

[15] Zawada AM Rogacev KS Rotter B Winter P Marell RR Fliser D. SuperSAGE evidence for CD14^++^CD16^+^ monocytes as a third monocyte subset. Blood (2011) 118:e50–61. 10.1182/blood-2011-01-326827 21803849

[16] Auffray C Sieweke MH Geissmann F. Blood monocytes: development, heterogeneity, and relationship with dendritic cells. Annu Rev Immunol. (2009) 27:669–92. 10.1146/annurev.immunol.021908.132557 19132917

[17] Nahrendorf M Swirski FK Aikawa E Stangenberg L Wurdinger T Figueiredo JL. The healing myocardium sequentially mobilizes two monocyte subsets with divergent and complementary functions. J Exp Med. (2007) 204:3037–47. 10.1084/jem.20070885 18025128 PMC2118517

[18] Olingy CE San Emeterio CL Ogle ME Krieger JR Bruce AC Pfau DD. Non-classical monocytes are biased progenitors of wound healing macrophages during soft tissue injury. Sci Rep. (2017) 7:447. 10.1038/s41598-017-00477-1 28348370 PMC5428475

[19] Hussen J Schuberth HJ. Heterogeneity of bovine peripheral blood monocytes. Front Immunol. (2017) 8:1875. 10.3389/fimmu.2017.01875 29312348 PMC5742132

[20] Grage-Griebenow E Flad HD Ernst M. Heterogeneity of human peripheral blood monocyte subsets. J Leukoc Biol. (2001) 69:11–20. 10.1189/jlb.69.1.11 11200054

[21] Hussen J Düvel A Sandra O Smith D Sheldon IM Zieger P. Phenotypic and functional heterogeneity of bovine blood monocytes. PLoS ONE (2013) 8:e71502. 10.1371/journal.pone.0071502 23967219 PMC3743816

[22] Corripio-Miyar Y Hope J McInnes CJ Wattegedera SR Jensen K Pang Y. Phenotypic and functional analysis of monocyte populations in cattle peripheral blood identifies a subset with high endocytic and allogeneic T-cell stimulatory capacity. Vet Res. (2015) 46:112. 10.1186/s13567-015-0246-4 26407849 PMC4582714

[23] Summerfield A Auray G Ricklin M. Comparative dendritic cell biology of veterinary mammals. Annu Rev Anim Biosci. (2015) 3:533–57. 10.1146/annurev-animal-022114-111009 25387110

[24] Auray G Keller I Python S Gerber M Bruggmann R Ruggli N. Characterization and transcriptomic analysis of porcine blood conventional and plasmacytoid dendritic cells reveals striking species-specific differences. J Immunol. (2016) 197:4791–806. 10.4049/jimmunol.1600672 27837108

[25] McKeever DJ MacHugh ND Goddeeris BM Awino E Morrison WI. Bovine afferent lymph veiled cells differ from blood monocytes in phenotype and accessory function. J Immunol. (1991) 147:3703–9. 1682381

[26] McKeever DJ Awino E Morrison WI. Afferent lymph veiled cells prime CD4^+^ T cell responses *in vivo*. Eur J Immunol. (1992) 22:3057–61. 10.1002/eji.1830221205 1359968

[27] Howard CJ Sopp P Brownlie J Kwong LS Parsons KR Taylor G. Identification of two distinct populations of dendritic cells in afferent lymph that vary in their ability to stimulate T cells. J Immunol. (1997) 159:5372–82. 9548477

[28] Howard CJ Brooke GP Werling D Sopp P Hope JC Parsons KR. Dendritic cells in cattle: phenotype and function. Vet Immunol Immunopathol. (1999) 72:119–24. 10614501 10.1016/s0165-2427(99)00124-5

[29] Gliddon DR Hope JC Brooke GP Howard CJ. DEC-205 expression on migrating dendritic cells in afferent lymph. Immunology (2004) 111:262–72. 10.1111/j.0019-2805.2004.01820.x 15009426 PMC1782417

[30] Reid E Juleff N Gubbins S Prentice H Seago J Charleston B. Bovine plasmacytoid dendritic cells are the major source of type I interferon in response to foot-and-mouth disease virus *in vitro* and *in vivo*. J Virol. (2011) 85:4297–308. 10.1128/jvi.02495-10 21307187 PMC3126242

[31] Werling D Hope JC Siddiqui N Widdison S Russell C Sopp P. Subset-specific expression of toll-like receptors by bovine afferent lymph dendritic cells. Front Vet Sci. (2017) 4:44. 10.3389/fvets.2017.00044 28421187 PMC5376590

[32] Renjifo X Howard C Kerkhofs P Denis M Urbain J Moser M. Purification and characterization of bovine dendritic cells from peripheral blood. Vet Immunol Immunopathol. (1997) 60:77–88. 9533268 10.1016/s0165-2427(97)00092-5

[33] Miyazawa K Aso H Honda M Kido T Minashima T Kanaya T. Identification of bovine dendritic cell phenotype from bovine peripheral blood. Res Vet Sci. (2006) 81:40–5. 10.1016/j.rvsc.2005.09.003 16253299

[34] Gibson A Miah S Griebel P Brownlie J Werling D. Identification of a lineage negative cell population in bovine peripheral blood with the ability to mount a strong type I interferon response. Dev Comp Immunol. (2012) 36:332–41. 10.1016/j.dci.2011.05.002 21663757

[35] Sei JJ Ochoa AS Bishop E Barlow JW Golde WT. Phenotypic, ultra-structural, and functional characterization of bovine peripheral blood dendritic cell subsets. PLoS ONE (2014) 9:e109273. 10.1371/journal.pone.0109273 25295753 PMC4190170

[36] Li K Wei G Cao Y Li D Li P Zhang J. The identification and distribution of cattle XCR1 and XCL1 among peripheral blood cells: new insights into the design of dendritic cells targeted veterinary vaccine. PLoS ONE (2017) 12:e0170575. 10.1371/journal.pone.0170575 28129380 PMC5271332

[37] Zhuang T Urakawa M Sato H Sato Y Taguchi T Umino T. Phenotypic and functional analysis of bovine peripheral blood dendritic cells before parturition by a novel purification method. Anim Sci J. (2018) 89:1011–9. 10.1111/asj.13014 29708291 PMC6055732

[38] Howard CJ Sopp P Brownlie J Parsons KR Kwong LS Collins RA Afferent lymph veiled cells stimulate proliferative responses in allogeneic CD4^+^ and CD8^+^ T cells but not gamma delta TCR^+^ T cells. Immunology (1996) 88:558–64. 8881757 10.1046/j.1365-2567.1996.d01-680.x PMC1456644

[39] González-Cano P Arsic N Popowych YI Griebel PJ. Two functionally distinct myeloid dendritic cell subpopulations are present in bovine blood. Dev Comp Immunol. (2014) 44:378–88. 10.1016/j.dci.2014.01.014 24502939

[40] Vu Manh TP Elhmouzi-Younes J Urien C Ruscanu S Jouneau L Bourge M Defining mononuclear phagocyte subset homology across several distant warm-blooded vertebrates through comparative transcriptomics. Front Immunol. (2015) 6:299 10.3389/fimmu.2015.00299 26150816 PMC4473062

[41] Ziegler A Marti E Summerfield A Baumann A. Identification and characterization of equine blood plasmacytoid dendritic cells. Dev Comp Immunol. (2016) 65:352–7. 10.1016/j.dci.2016.08.005 27524460

[42] Guzylack-Piriou L Alves MP McCullough KC Summerfield A. Porcine Flt3 ligand and its receptor: generation of dendritic cells and identification of a new marker for porcine dendritic cells. Dev Comp Immunol. (2010) 34:455–64. 10.1016/j.dci.2009.12.006 20015454

[43] Wong KL Tai JJ Wong WC Han H Sem X Yeap WH. Gene expression profiling reveals the defining features of the classical, intermediate, and nonclassical human monocyte subsets. Blood (2011) 118:e16–31. 10.1182/blood-2010-12-326355 21653326

[44] Ingersoll MA Spanbroek R Lottaz C Gautier EL Frankenberger M Hoffmann R. Comparison of gene expression profiles between human and mouse monocyte subsets. Blood (2010) 115:e10–9. 10.1182/blood-2009-07-235028 19965649 PMC2810986

[45] Fairbairn L Kapetanovic R Beraldi D Sester DP Tuggle CK Archibald AL. Comparative analysis of monocyte subsets in the pig. J Immunol. (2013) 190:6389–96. 10.4049/jimmunol.1300365 23667115

[46] Miller JA Cai C Langfelder P Geschwind DH Kurian SM Salomon DR. Strategies for aggregating gene expression data: the collapseRows R function. BMC Bioinformatics (2011) 12:322. 10.1186/1471-2105-12-322 21816037 PMC3166942

[47] Lottaz C Yang X Scheid S Spang R. OrderedList—A bioconductor package for detecting similarity in ordered gene lists. Bioinformatics (2006) 22:2315–6. 10.1093/bioinformatics/btl385 16844712

[48] Karsunky H Merad M Cozzio A Weissman IL Manz MG. Flt3 ligand regulates dendritic cell development from Flt3^+^ lymphoid and myeloid-committed progenitors to Flt3^+^ dendritic cells *in vivo*. J Exp Med. (2003) 198:305–13. 10.1084/jem.20030323 12874263 PMC2194067

[49] Schmid MA Kingston D Boddupalli S Manz MG. Instructive cytokine signals in dendritic cell lineage commitment. Immunol Rev. (2010) 234:32–44. 10.1111/j.0105-2896.2009.00877.x 20193010

[50] Lee J Zhou YJ Ma W Zhang W Aljoufi A Luh T Lineage specification of human dendritic cells is marked by IRF8 expression in hematopoietic stem cells and multipotent progenitors. Nat Immunol. (2017) 18:877–88. 10.1038/ni.3789 28650480 PMC5743223

[51] Parsons KR Bembridge G Sopp P Howard CJ. Studies of monoclonal antibodies identifying two novel bovine lymphocyte antigen differentiation clusters: workshop clusters (WC) 6 and 7. Vet Immunol Immunopathol. (1993) 39:187–92. 8310644 10.1016/0165-2427(93)90180-c

[52] Collin M Bigley V. Human dendritic cell subsets: an update. Immunology (2018) 154:3–20. 10.1111/imm.12888 29313948 PMC5904714

[53] Dutertre CA Wang LF Ginhoux F. Aligning *bona fide* dendritic cell populations across species. Cell Immunol. (2014) 291:3–10. 10.1016/j.cellimm.2014.08.006 25262488

[54] Robbins SH Walzer T Dembélé D Thibault C Defays A Bessou G. Novel insights into the relationships between dendritic cell subsets in human and mouse revealed by genome-wide expression profiling. Genome Biol. (2008) 9:R17. 10.1186/gb-2008-9-1-r17 18218067 PMC2395256

[55] See P Dutertre CA Chen J Günther P McGovern N Irac SE. Mapping the human DC lineage through the integration of high-dimensional techniques. Science (2017) 356:eaag3009. 10.1126/science.aag3009 28473638 PMC7611082

[56] Contreras V Urien C Guiton R Alexandre Y Vu Manh TP Andrieu T. Existence of CD8alpha-like dendritic cells with a conserved functional specialization and a common molecular signature in distant mammalian species. J Immunol. (2010) 185:3313–25. 10.4049/jimmunol.1000824 20702727

[57] Crozat K Guiton R Contreras V Feuillet V Dutertre CA Ventre E. The XC chemokine receptor 1 is a conserved selective marker of mammalian cells homologous to mouse CD8α^+^ dendritic cells. J Exp Med. (2010) 207:1283–92. 10.1084/jem.20100223 20479118 PMC2882835

[58] Heger L Balk S Lühr JJ Heidkamp GF Lehmann CHK Hatscher L. CLEC10A is a specific marker for human CD1c^+^ dendritic cells and enhances their toll-like receptor 7/8-induced cytokine secretion. Front Immunol. (2018) 9:744. 10.3389/fimmu.2018.00744 29755453 PMC5934495

[59] Zhuang Y Mwangi W Brown WC Davis WC Hope JC Palmer GH. Characterization of a phenotypically unique population of CD13^+^ dendritic cells resident in the spleen. Clin Vaccine Immunol. (2006) 13:1064–9. 10.1128/cvi.00178-06 16960120 PMC1563577

[60] Fries P Popowych YI Guan LL Beskorwayne T Potter A Babiuk L. Mucosal dendritic cell subpopulations in the small intestine of newborn calves. Dev Comp Immunol. (2011) 35:1038–49. 10.1016/j.dci.2011.04.003 21527286

[61] Edwards AD Diebold SS Slack EM Tomizawa H Hemmi H Kaisho T Toll-like receptor expression in murine DC subsets: lack of TLR7 expression by CD8alpha^+^ DC correlates with unresponsiveness to imidazoquinolines. Eur J Immunol. (2003) 33:827–33. 10.1002/eji.200323797 12672047

[62] Hémont C Neel A Heslan M Braudeau C Josien R. Human blood mDC subsets exhibit distinct TLR repertoire and responsiveness. J Leukoc Biol. (2013) 93:599–609. 10.1189/jlb.0912452 23341538

[63] Diacovo TG Blasius AL Mak TW Cella M Colonna M. Adhesive mechanisms governing interferon-producing cell recruitment into lymph nodes. J Exp Med. (2005) 202:687–96. 10.1084/jem.20051035 16147979 PMC2212867

[64] Randolph GJ Ochando J Partida-Sanchez S. Migration of dendritic cell subsets and their precursors. Annu Rev Immunol. (2008) 26:293–316. 10.1146/annurev.immunol.26.021607.090254 18045026

[65] Jakubzick C Gautier EL Gibbings SL Sojka DK Schlitzer A Johnson TE. Minimal differentiation of classical monocytes as they survey steady-state tissues and transport antigen to lymph nodes. Immunity (2013) 39:599–610. 10.1016/j.immuni.2013.08.007 24012416 PMC3820017

[66] Vermi W Riboldi E Wittamer V Gentili F Luini W Marrelli S. Role of ChemR23 in directing the migration of myeloid and plasmacytoid dendritic cells to lymphoid organs and inflamed skin. J Exp Med. (2005) 201:509–15. 10.1084/jem.20041310 15728234 PMC2213064

[67] Iberg CA Jones A Hawiger D. Dendritic cells as inducers of peripheral tolerance. Trends Immunol. (2017) 38:793–804. 10.1016/j.it.2017.07.007 28826942 PMC5669994

[68] Audiger C Rahman MJ Yun TJ Tarbell KV Lesage S. The importance of dendritic cells in maintaining immune tolerance. J Immunol. (2017) 198:2223–31. 10.4049/jimmunol.1601629 28264998 PMC5343761

[69] Hadeiba H Lahl K Edalati A Oderup C Habtezion A Pachynski R. Plasmacytoid dendritic cells transport peripheral antigens to the thymus to promote central tolerance. Immunity (2012) 36:438–50. 10.1016/j.immuni.2012.01.017 22444632 PMC3315699

[70] Brewitz A Eickhoff S Dähling S Quast T Bedoui S Kroczek RA. CD8^+^ T cells orchestrate pDC-XCR1^+^ dendritic cell spatial and functional cooperativity to optimize priming. Immunity (2017) 46:205–19. 10.1016/j.immuni.2017.01.003 28190711 PMC5362251

[71] Lippitsch A Chukovetskyi Y Baal N Bein G Hackstein H. Unique high and homogenous surface expression of the transferrin receptor CD71 on murine plasmacytoid dendritic cells in different tissues. Cell Immunol. (2017) 316:41–52. 10.1016/j.cellimm.2017.03.005 28372797

[72] Sancho D Joffre OP Keller AM Rogers NC Martínez D Hernanz-Falcón P. Identification of a dendritic cell receptor that couples sensing of necrosis to immunity. Nature (2009) 458:899–903. 10.1038/nature07750 19219027 PMC2671489

[73] Gutiérrez-Martínez E Planès R Anselmi G Reynolds M Menezes S Adiko AC. Cross-presentation of cell-associated antigens by MHC class I in dendritic cell subsets. Front Immunol. (2015) 6:363. 10.3389/fimmu.2015.00363 26236315 PMC4505393

[74] Lau CM Tiniakou I Perez OA Kirkling ME Yap GS Hock H. Transcription factor Etv6 regulates functional differentiation of cross-presenting classical dendritic cells. J Exp Med. (2018) 215:2265–78. 10.1084/jem.20172323 30087163 PMC6122974

[75] Clement M Fornasa G Guedj K Ben Mkaddem S Gaston AT Khallou-Laschet J. CD31 is a key coinhibitory receptor in the development of immunogenic dendritic cells. Proc Natl Acad Sci USA. (2014) 111:E1101–10. 10.1073/pnas.1314505111 24616502 PMC3970499

[76] Cabezón R Carrera-Silva EA Flórez-Grau G Errasti AE Calderón-Gómez E Lozano JJ. MERTK as negative regulator of human T cell activation. J Leukoc Biol. (2015) 97:751–60. 10.1189/jlb.3A0714-334R 25624460 PMC4370049

[77] Nakano H Lin KL Yanagita M Charbonneau C Cook DN Kakiuchi T. Blood-derived inflammatory dendritic cells in lymph nodes stimulate acute T helper type 1 immune responses. Nat Immunol. (2009) 10:394–402. 10.1038/ni.1707 19252492 PMC2668134

[78] Wynn TA Vannella KM. Macrophages in tissue repair, regeneration, and fibrosis. Immunity (2016) 44:450–62. 10.1016/j.immuni.2016.02.015 26982353 PMC4794754

[79] Ziegler-Heitbrock L. Monocyte subsets in man and other species. Cell Immunol. (2014) 289:135–9. 10.1016/j.cellimm.2014.03.019 24791698

[80] Hur J Choi JI Yun JY Yoon CH Jang JH Im SG. Highly angiogenic CXCR4^+^CD31^+^ monocyte subset derived from 3D culture of human peripheral blood. Biomaterials (2013) 34:1929–41. 10.1016/j.biomaterials.2012.11.015 23267826

[81] Zizzo G Hilliard BA Monestier M Cohen PL. Efficient clearance of early apoptotic cells by human macrophages requires M2c polarization and MerTK induction. J Immunol. (2012) 189:3508–20. 10.4049/jimmunol.1200662 22942426 PMC3465703

[82] Thielens NM Tedesco F Bohlson SS Gaboriaud C Tenner AJ. C1q: a fresh look upon an old molecule. Mol Immunol. (2017) 89:73–83. 10.1016/j.molimm.2017.05.025 28601358 PMC5582005

[83] Moreno S Alvarez B Poderoso T Revilla C Ezquerra A Alonso F. Porcine monocyte subsets differ in the expression of CCR2 and in their responsiveness to CCL2. Vet Res. (2010) 41:76. 10.1051/vetres/2010048 20670605 PMC2941139

[84] Harman AN Bye CR Nasr N Sandgren KJ Kim M Mercier SK. Identification of lineage relationships and novel markers of blood and skin human dendritic cells. J Immunol. (2013) 190:66–79. 10.4049/jimmunol.1200779 23183897

[85] Hedges JF Kimmel E Snyder DT Jerome M Jutila MA. Solute carrier 11A1 is expressed by innate lymphocytes and augments their activation. J Immunol. (2013) 190:4263–73. 10.4049/jimmunol.1200732 23509347 PMC3622125

[86] Singh N Gedda MR Tiwari N Singh SP Bajpai S Singh RK. Solute carrier protein family 11 member 1 (Slc11a1) activation efficiently inhibits *Leishmania donovani* survival in host macrophages. J Parasit Dis. (2017) 41:671–7. 10.1007/s12639-016-0864-4 28848257 PMC5555910

[87] Blasius AL Arnold CN Georgel P Rutschmann S Xia Y Lin P. Slc15a4, AP-3, and Hermansky-Pudlak syndrome proteins are required for Toll-like receptor signaling in plasmacytoid dendritic cells. Proc Natl Acad Sci USA. (2010) 107:19973–8. 10.1073/pnas.1014051107 21045126 PMC2993408

[88] Blasius AL Krebs P Sullivan BM Oldstone MB Popkin DL. Slc15a4, a gene required for pDC sensing of TLR ligands, is required to control persistent viral infection. PLoS Pathog. (2012) 8:e1002915. 10.1371/journal.ppat.1002915 23028315 PMC3441671

[89] Skazik C Heise R Bostanci Ö Paul N Denecke B Joussen S. Differential expression of influx and efflux transport proteins in human antigen presenting cells. Exp Dermatol. (2008) 17:739–47. 10.1111/j.1600-0625.2008.00745.x 18557925

[90] Cekic C Linden J. Purinergic regulation of the immune system. Nat Rev Immunol. (2016) 16:177–92. 10.1038/nri.2016.4 26922909

[91] Shin A Toy T Rothenfusser S Robson N Vorac J Dauer M. P2Y receptor signaling regulates phenotype and IFN-α secretion of human plasmacytoid dendritic cells. Blood (2008) 111:3062–9. 10.1182/blood-2007-02-071910 17993619

[92] Jones EL Wee JL Demaria MC Blakeley J Ho PK Vega-Ramos J. Dendritic cell migration and antigen presentation are coordinated by the opposing functions of the tetraspanins CD82 and CD37. J Immunol. (2016) 196:978–87. 10.4049/jimmunol.1500357 26729805

[93] Zuidscherwoude M Worah K van der Schaaf A Buschow SI van Spriel AB. Differential expression of tetraspanin superfamily members in dendritic cell subsets. PLoS ONE (2017) 12:e0184317. 10.1371/journal.pone.0184317 28880937 PMC5589240

[94] Reyes R Cardeñes B Machado-Pineda Y Cabañas C. Tetraspanin CD9: a key regulator of cell adhesion in the immune system. Front Immunol. (2018) 9:863. 10.3389/fimmu.2018.00863 29760699 PMC5936783

[95] Feinstein J Ramkhelawon B. Netrins & semaphorins: novel regulators of the immune response. Biochim Biophys Acta (2017) 1863:3183–9. 10.1016/j.bbadis.2017.09.010 28918114 PMC6238647

[96] Nakagawa Y Takamatsu H Okuno T Kang S Nojima S Kimura T. Identification of semaphorin 4B as a negative regulator of basophil-mediated immune responses. J Immunol. (2011) 186:2881–8. 10.4049/jimmunol.1003485 21270411

[97] Kumanogoh A Marukawa S Suzuki K Takegahara N Watanabe C Ch'ng E. Class IV semaphorin Sema4A enhances T-cell activation and interacts with Tim-2. Nature (2002) 419:629–33. 10.1038/nature01037 12374982

[98] Delgoffe GM Woo SR Turnis ME Gravano DM Guy C Overacre AE. Stability and function of regulatory T cells is maintained by a neuropilin-1-semaphorin-4a axis. Nature (2013) 501:252–6. 10.1038/nature12428 23913274 PMC3867145

[99] Kumanogoh A Watanabe C Lee I Wang X Shi W Araki H. Identification of CD72 as a lymphocyte receptor for the class IV semaphorin CD100: a novel mechanism for regulating B cell signaling. Immunity (2000) 13:621–31. 10.1016/S1074-7613(00)00062-5 11114375

[100] Kumanogoh A Suzuki K Ch'ng E Watanabe C Marukawa S Takegahara N. Requirement for the lymphocyte semaphorin, CD100, in the induction of antigen-specific T cells and the maturation of dendritic cells. J Immunol. (2002) 169:1175–81. 10.4049/jimmunol.169.3.1175 12133937

[101] Holmes S Downs AM Fosberry A Hayes PD Michalovich D Murdoch P. Sema7A is a potent monocyte stimulator. Scand J Immunol. (2002) 56:270–5. 10.1046/j.1365-3083.2002.01129.xs 12193228

